# Root Canal Wall Adaptation of a Novel Filling Material: A Micro-CT Ex Vivo Evaluation

**DOI:** 10.3390/dj14080501

**Published:** 2026-08-08

**Authors:** Peter Kiefner, Mirela Gules, Marina Bunea, Michael Ban

**Affiliations:** 1 Praxis für Zahnheilkunde/Endodontologie und Dentale Traumatologie, 70178 Stuttgart, Germany; mirelutza3@yahoo.com; 2 Research and Development Center for Thermoset Matrix Composites, “Dunarea de Jos” University of Galati, 111 Domneasca, 800201 Galati, Romania; bunea.marina@usch.md; 3 “Bogdan Petriceicu Hasdeu” University Center of Cahul, Technical University of Moldova, 1 Piata Independentei, MD-3909 Cahul, Moldova; 4 Institut für Allgemeine Mechanik, RWTH Aachen University, Eilfschornsteinstraße 18, 52062 Aachen, Germany; ban@iam.rwth-aachen.de

**Keywords:** root canal treatment, root canal filling, filling material, adaptation

## Abstract

**Background/Objectives**: This study evaluated the adaptation of a novel filling material, Odne^®^Fill (ODNE, Dübendorf, Switzerland), to instrumented root canal walls using micro-computed tomography. **Methods**: Fourteen extracted human molars (six maxillary and eight mandibular), comprising a total of 32 root canals, were included in this study. Following preparation, all root canals were filled with Odne^®^Fill according to the manufacturer’s instructions. The extent of the root canal area coverage by the novel filling material was assessed using both periapical radiographs and micro-CT. The filling material adaptation to the canal walls was quantified through image segmentation and statistically analyzed. **Results**: The percentage of canal wall coverage ranged from 93.31% to 99.98%. The mean coverage rate was 97.63% (standard deviation: 1.70%), with a median value of 98.23%, indicating a high degree of adaptation of the filling material to the ex vivo prepared canal walls. **Conclusions**: Within the limitations of this ex vivo study, Odne^®^Fill demonstrated high adaptation values to the prepared root canal walls. These findings should be interpreted with caution. Further comparative studies and long-term investigations are required to determine the clinical significance and performance of the studied material under in vivo conditions.

## 1. Introduction

Root canal obturation represents an important step in endodontic treatment and is performed following chemomechanical preparation and disinfection of the root canal system [1,2]. Its primary objective is to fill the prepared canal space and thereby reduce the risk of reinfection. Over the years, a wide range of obturation materials and techniques have been introduced for this purpose [3,4].

Conventional root canal filling is mainly using a gutta-percha core in combination with a sealer placed between the obturation material and the instrumented canal walls [5]. Gutta-percha remains the most widely used core material because of its favorable physical and chemical stability as well as its ability to be thermoplasticized [6]. Among the variety of obturation techniques [7,8] cold lateral compaction and warm vertical compaction of gutta-percha are the most commonly employed in clinical practice [9,10].

Cold lateral compaction has long been regarded as the standard obturation technique and has frequently served as a reference method in endodontic research. However, this technique has several limitations, including incomplete adaptation of the filling material to the canal walls [11] and the generation of high compaction forces during this procedure [12].

Warm vertical compaction has been proposed as an alternative approach because thermoplasticized gutta-percha may achieve improved adaptation to the prepared root canal walls and more effectively fill anatomical irregularities [13]. Nevertheless, this technique is highly operator-dependent and more time-consuming than cold lateral compaction [14,15].

Despite its long-standing clinical use, gutta-percha does not chemically bond to dentine and exhibits no adhesion to the canal wall [16]. This limitation has stimulated the research and development of alternative obturation concepts and materials aimed at improving the adaptation to the prepared root canal surface and enhancing the quality of the root canal filling [17].

The Resilon^®^/Epiphany obturation system (Pentron Clinical Technologies, Wallingford, CT, USA) was developed and recommended as a root canal filling system to overcome the limitations of gutta-percha. Resilon^®^—a polycaprolactone polymer—showed a chemical bonding to the canal-wall dentin when used in combination with Epiphany^®^ sealer [18]. This material is prone to hydrolysis by enzymes, mechanical load and wear [19].

The degradation rate is higher compared with the gutta-percha/sealer combination and has a major correlation with non-healed endodontic cases [20]. In the long term the Resilon^®^ system demonstrated an increased fluid flow due to degradation processes [21].

In this context, Odne^®^Fill (ODNE, Dübendorf, Switzerland), a low-viscosity light-curable hydrophilic gel, has recently been introduced as a novel root canal filling material [22]. According to the manufacturer Odne^®^Fill is a light-curing, injectable single-component root canal filling material. It is a highly stable hydrogel, a material class characterized by biocompatibility [23] and hydrophilicity. Due to these properties and the water-like viscosity in its uncured state, the material can easily penetrate complex endodontic structures, such as isthmuses, apical deltas C-shaped canals, etc. The material has been specially formulated to mechanically create a very tight interface with the root canal dentin in its cured state.

A minimum preparation of the root canal is needed to inject and light-cure the material in the root canal. Its delivery system includes a fine and flexible cannula (diameter 190 µm) and a dedicated fiber-optic tip (diameter 220 µm) for intracanal light curing. For a proper obturation of the root canal with this material, there is no need of compaction forces or thermal treatment. The cannula is inserted into the root canal at working length and the material is then gently applied from the apex. The material is light-blue in its uncured state. Odne^®^Fill is then instantly cured through activation of the Odne^®^Cure micro-laser. After light-curing the material becomes transparent. Odne^®^Fill is cured in two steps: first, curing at working length −3 mm to −5 mm to seal above the apex. Second, curing above the root canal entry finalizes sealing the canal.

The material provides a gap-free root canal sealing and is compatible with most prosthodontic materials and procedures for the final restoration of the teeth. In case of orthograde retreatment, Odne^®^Fill can be easily removed from the root canal space due to its pulp-like mechanical properties.

The root canal fillings technique using this material fundamentally differs from conventional gutta-percha-based techniques. The adaptation of Odne^®^Fill to the prepared root canal wall warrants evaluation. Micro-computed tomography (micro-CT) provides a non-destructive three-dimensional method for evaluation of root canal fillings [24] and enables quantification of the extent of canal wall coverage by the filling material. Therefore, the aim of the present ex vivo study was to evaluate the adaptation of ODNE^®^Fill to prepared root canal walls using micro-computed tomography.

## 2. Materials and Methods

### 2.1. Sample Size Calculation and Statistical Analysis

Sample size estimation was performed using G*Power 3.1 for macOS (Heinrich Heine University Düsseldorf, Düsseldorf, Germany) based on data reported in previous studies [25,26]. Assuming a significance level of α = 0.05 and a statistical power of 95%, the minimum required sample size was calculated to be 29 canals. To account for potential variability, a total of 32 root canals were included in the present ex vivo study.

The extent of the surface coverage between the obturation material and the prepared root canal walls was quantified and analyzed using one-sample *t*-test in MATLAB 25.2 (Statistics and Machine Learning Toolbox, MathWorks, Natick, MA, USA). Descriptive statistics, including mean, standard deviation, median, minimum and maximum values were calculated.

In addition to descriptive statistics, the measured surface coverage values were compared with two predefined exploratory analytical reference levels of 95% and 97%, which were used to contextualize the distribution of the measured canal wall coverage values. These values were not intended to represent validated clinical thresholds, but served only as analytical reference levels for descriptive interpretation. For each comparison, one-sample *t*-tests were performed and *p*-values, confidence intervals, and test statistics were recorded. This approach enabled a quantitative description of the adaptation of the obturation material to the root canal walls and provided an exploratory comparison of the observed coverage values with predefined analytical reference levels.

No equivalence or non-inferiority testing was performed in the present study. Therefore, the one-sample *t*-test results were interpreted only as exploratory comparisons with predefined analytical reference levels and were not used to infer statistical equivalence, non-inferiority, clinical adequacy, or superiority.

### 2.2. Specimens and Canal Preparation

Human molars extracted for reasons unrelated to this study were collected and used in accordance with applicable ethical requirements. Ethical approval was not required because the study used anonymized extracted teeth. Six maxillary and eight mandibular molars without signs of root resorption, fractures, previous endodontic treatment, or other structural alterations were included. The extracted teeth were stored in a moist environment at body temperature until use. The crowns were removed at the level of the cemento-enamel junction using a diamond bur under water cooling to obtain standardized root specimens. A total of 32 root canals were subsequently prepared, cleaned, and dried according to the manufacturer’s instructions for the obturation system under investigation. The 32 root canals were distributed as follows: upper molar canals—4 mesio-buccal canals, 4 disto-buccal canals and 6 palatal canals. Lower molars: 6 mesio-buccal canals, 5 mesio-lingual canals and 7 distal canals.

### 2.3. Shaping and Filling Procedures

A size 0.08 stainless-steel K-file was inserted into each root canal until its tip became visible at the apical foramen. The working length was established 1.0 mm short of this point and was monitored throughout canal preparation using the integrated apex locator of the endodontic motor (X-Smart Pro Plus, Dentsply Sirona, Bensheim, Germany). The determined working length was subsequently verified by digital periapical radiography. All root canals exhibited moderate curvatures ranging from 10° to 30°.

Root canal preparation was performed using Reciproc Blue instruments (VDW, Munich, Germany) operated with an endodontic motor (X-Smart Pro Plus, Dentsply Sirona, Bensheim, Germany) according to the manufacturer’s recommended settings. The instruments were advanced apically in 3-mm increments until the predetermined working length was reached. In canals where the Reciproc Blue R25 instrument did not readily progress to the full working length, an R-Pilot file (VDW, Munich, Germany) was used to establish glide path and maintain canal patency.

After three apically directed pecking motions, the instrument was withdrawn and cleaned of the accumulated debris using a sponge soaked in 5.25% sodium hypochlorite mounted on an Interim Stand (VDW, Munich, Germany). The final apical preparation size corresponded to an ISO size 0.25 instrument. Throughout instrumentation, canals were irrigated alternately with 5.25% sodium hypochlorite and 17% EDTA.

Final irrigation was performed using the OdneClean^®^ hydrodynamic cavitation-based irrigation system equipped with single-use OdneClean^®^ tips (ODNE AG, Dübendorf, Switzerland). Due to their diameter of 190 µm, the irrigation tips could be inserted to the desired depth without difficulty. Distilled water was used as the irrigant according to the manufacturer’s instructions. The insertion depth and intracanal position of the irrigation tip were evaluated using the supplied silicone stop (Endostop).

Root canal obturation was performed using Odne^®^Fill (ODNE AG, Dübendorf, Switzerland; Lot No. 1352ED). Prior to material placement, the position of the flexible conical 33-gauge (33G) plastic cannula was adjusted by setting the Endostop rubber stop to 1.0 mm short of the previously determined working length. The single-use tips were then discarded.

**Figure 1:**
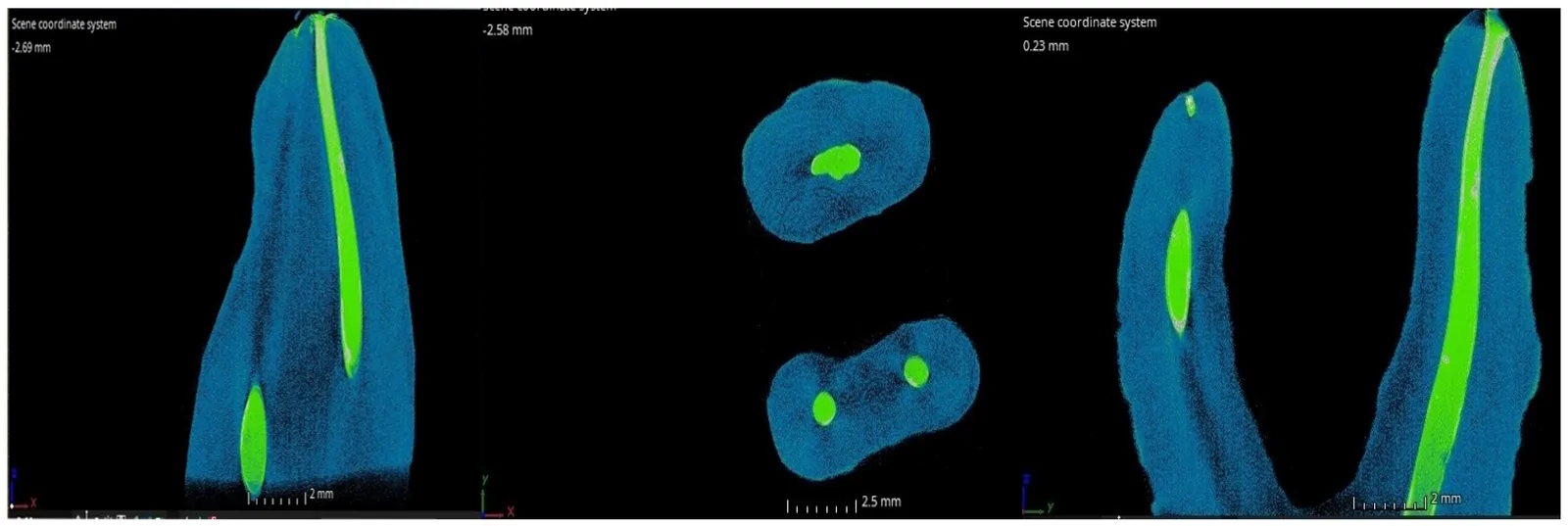

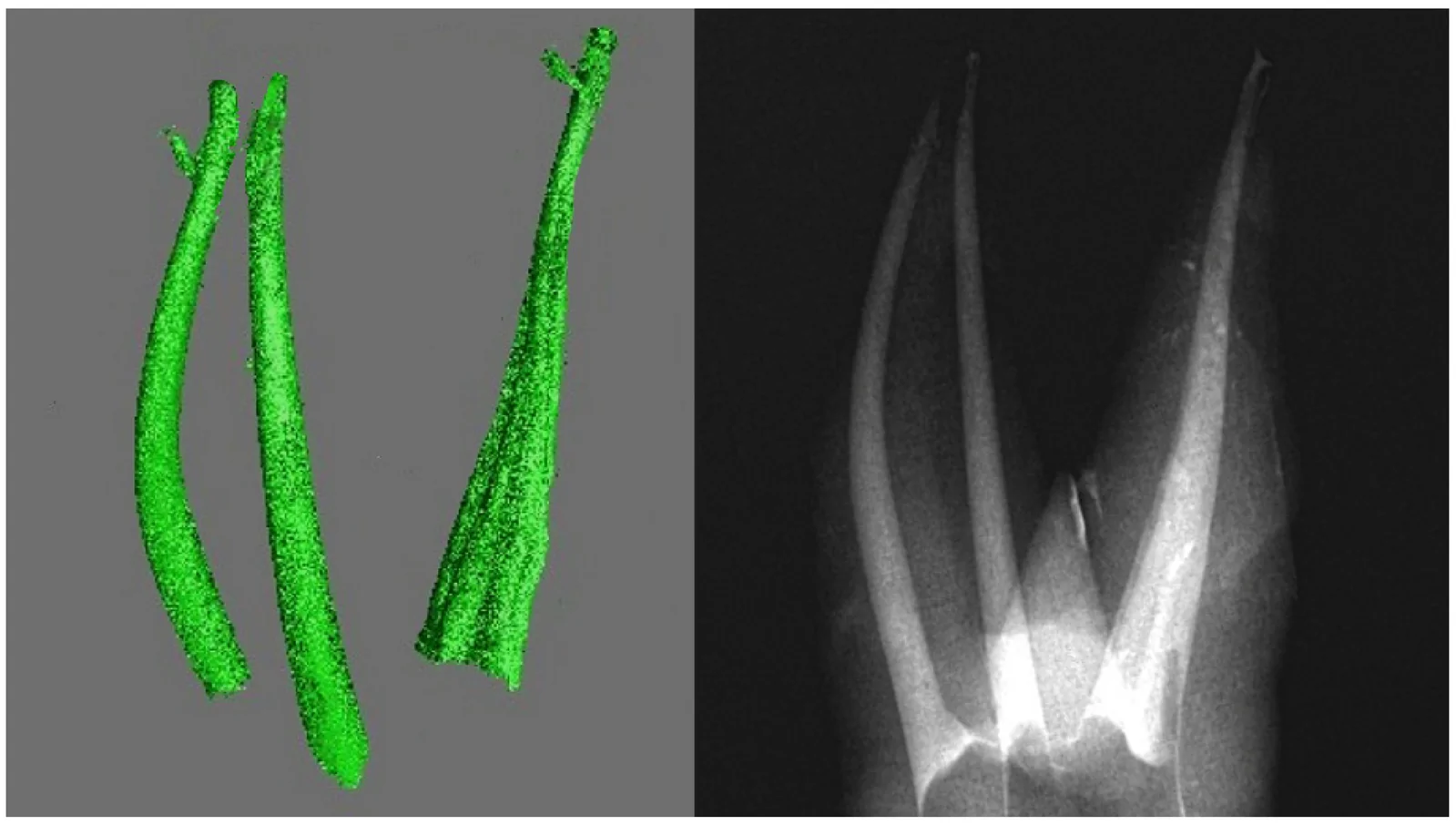
CT and radiographic image of the root canal filling.

After obturation all root canals were examined by digital periapical radiography and micro-CT to assess the extent of canal wall coverage achieved by the filling material (Figure 1).

### 2.4. CT Imaging and Image Construction

Micro-CT imaging was performed using a ProCon X-Ray Alpha device (ProCon X-Ray, Sarstedt, Germany) equipped with an XWT-225-TCNG X-ray tube (X-Ray WorX, Garbsen, Germany) and a Shad-o-Box 6K HS CMOS detector with a resolution of 49.5 µm (Teledyne DALSA, Waterloo, ON, Canada). All specimens were scanned at an acceleration voltage of 80 kV and a tube current of 120 µA corresponding to a power output of 9.6 W. For each scan, 1440 projection images were acquired over 360° with a rotation step of 0.25° and an exposure time of 1.2 s per projection. Images were acquired without filters at a geometric magnification of 6.96- to 9.157-fold, resulting in a voxel size ranging from 4.978 to 7.122 µm, depending on tooth size. The average scan time was approximately 45 min. Image reconstruction was performed using VGStudio MAX 2025.2 software (Volume Graphics, Heidelberg, Germany).

### 2.5. Determination of the Surface Ratio in Obturated Root Canals Using Image Segmentation

**Figure 2:**
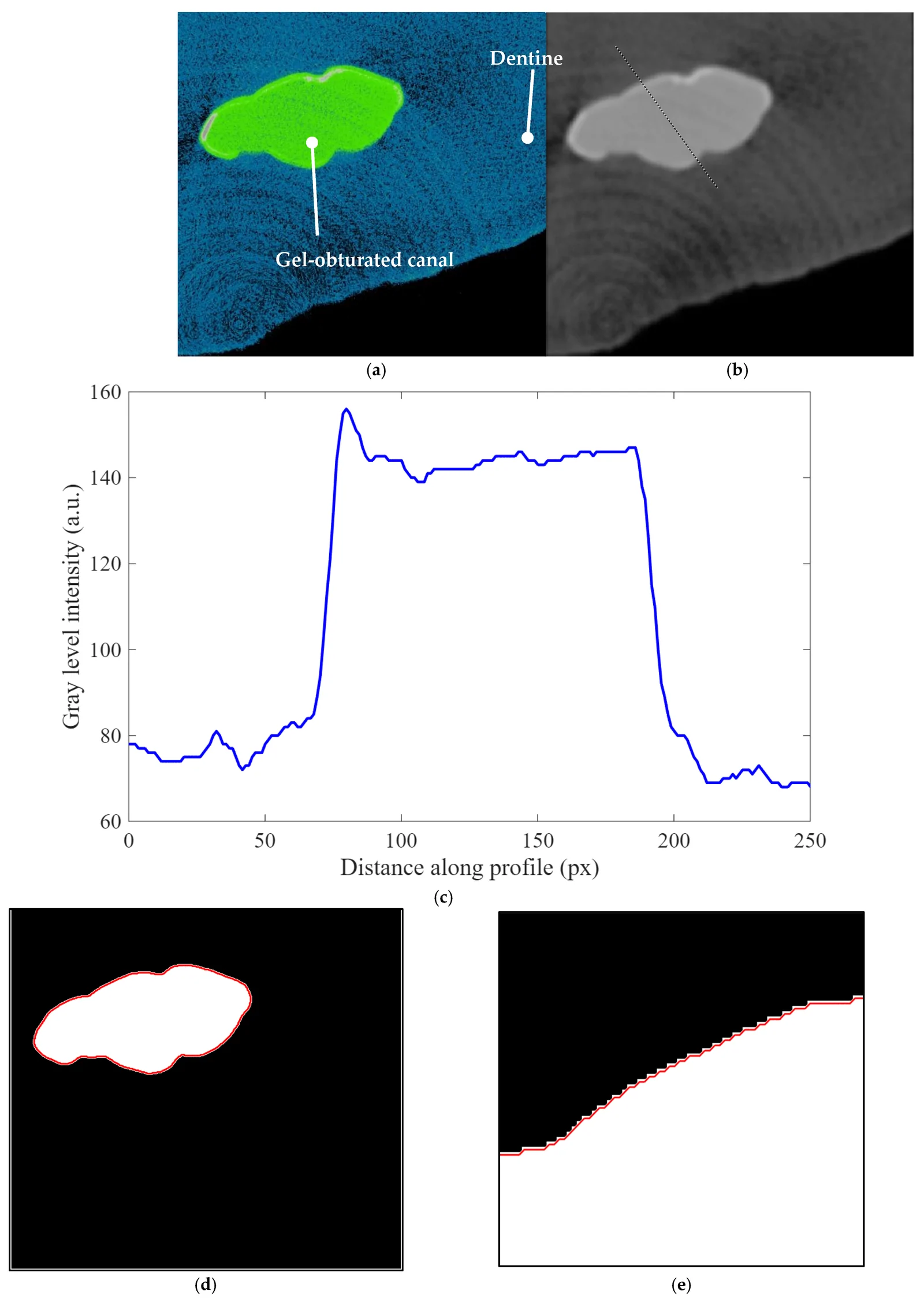
(**a**) RGB image. (**b**) Filtered grayscale image. Green area = obturation material in the root canal, Blue area = root dentine. (**c**) Plot of the pixel values along the line segment of grayscale image. (**d**) Red gel contour. (**e**) Gel contour limit, magnified section of (**d**).

Each coloured image (root canal filling—green area; canal wall dentine—blue area) of a micro-CT slice (Figure 2a) has been segmented primarily on the basis of grayscale intensity (Figure 2b).

To determine an appropriate segmentation threshold, the improfile function was used to extract grayscale intensity profiles along representative lines traversing the interface between the filling material and the root canal wall. Based on these profiles, fixed threshold values were established. Subsequently, a global intensity threshold was applied to the filtered grayscale images to segment and isolate pixels corresponding to the obturation material (Figure 2c).

The resulting binary mask was subsequently processed using a morphological hole-filling operation to eliminate internal voids arising from local intensity variations or partial-volume effects. This procedure ensured a continuous and anatomically plausible representation of the filling material within each image slice (Figure 2d,e). Figure 2e represents a part of the magnified gel contour shown in Figure 2d.

These slice-wise binary masks were used further for the volumetric reconstruction of the filling material throughout the shaped and filled root canal.

**Figure 3:**
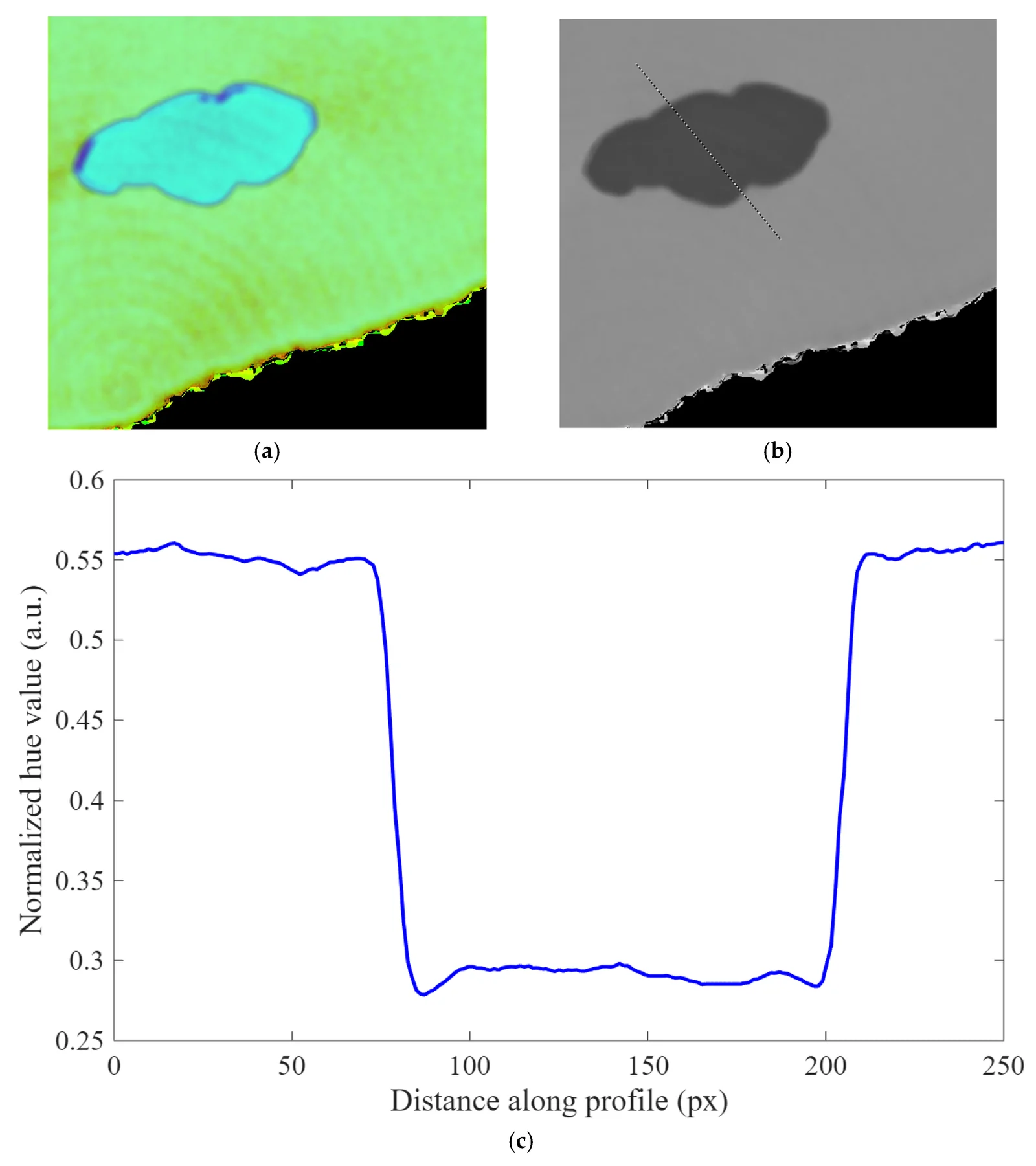
(**a**) HSV image. (**b**) Hue image. The root canal dentine = light green, the obturated root canal = light blue. (**c**) Plot of pixel values along the line segment of a hue image.

Hue saturation values (HSVs) and hue images were created (Figure 3a,b) allowing to plot the pixel values along a representative line segment in the hue image (Figure 3c).

**Figure 4:**
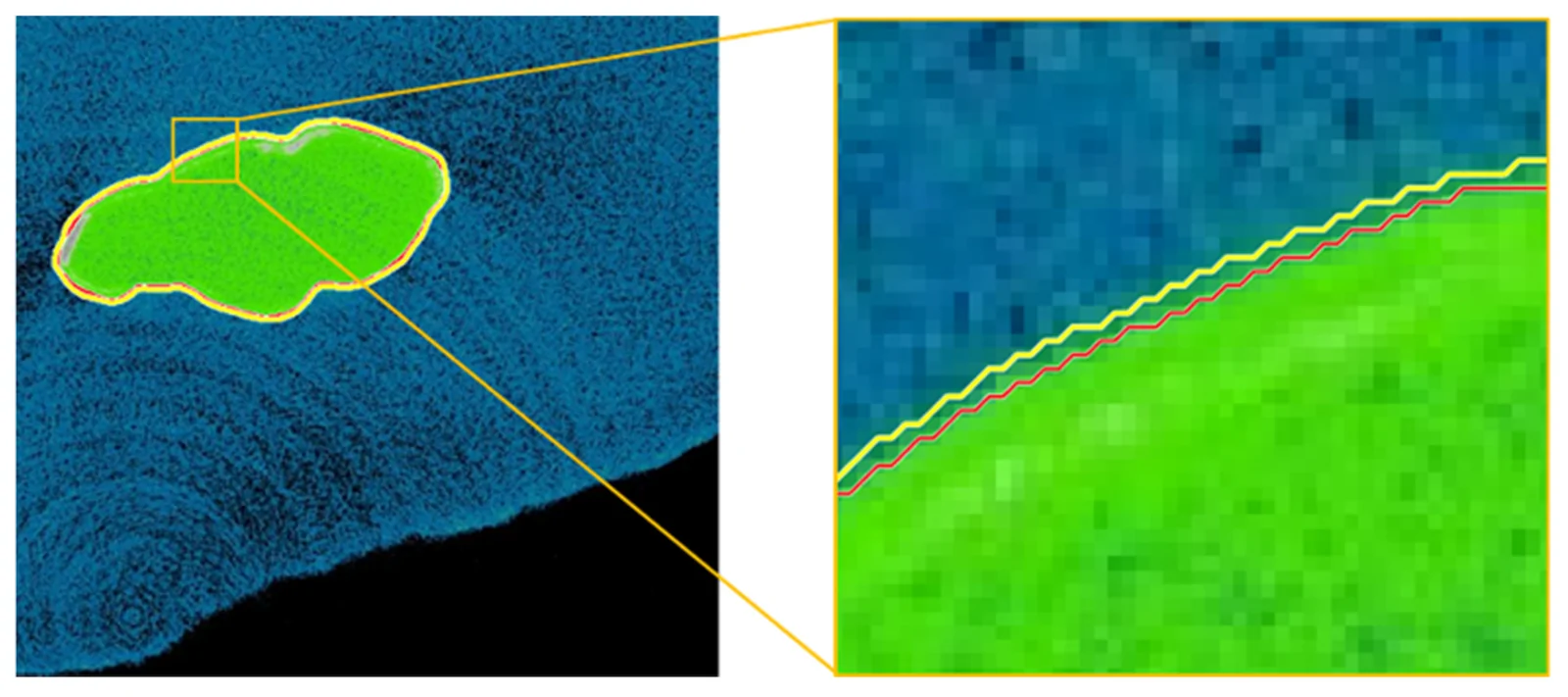
Interface between filling material and root canal wall. The square area shows a magnified detail view of the interface between the filling material and the root canal wall (the blue colour area represents root dentine, the green area is determined by the root canal filling materia. The yellow line represents the dentine wall outline, the red line the material outline—inbetween is the interface/adaptation area between material and shaped canal wall—this area was used for statistical analysis).

The initial region of interest comprised the entire canal cross-section including both filled and unfilled areas. To distinguish the filling material from voids within the canal, a second, more restrictive hue threshold was applied. This allowed identification of the uniform blue reference region corresponding to the intact canal background. By subtracting this strict blue mask from the broader canal region of interest (ROI), pixels representing color variations associated with the filling material or material interfaces were isolated. This differential segmentation strategy enabled reliable identification of the interface between the filling material and the root canal wall (Figure 4).

The segmented datasets of the obturation material and root canal space, obtained on a slice-by-slice basis, were subsequently used to quantify the canal wall surface coverage ratio for each root canal. This parameter represented the proportion of the prepared root canal surface in contact with the obturation material.

## 3. Results

The image segmentation procedure applied to the 32 filled root canals enabled quantitative assessment of surface coverage, defined as the adaptation of the filling material to the root canal wall. Overall, consistently high surface coverage values were observed across the investigated samples.

Surface coverage values ranged from 93.31% to 99.98%. The mean surface coverage was 97.63% (SD 1.70%), and the median was 98.23%. These findings indicate a high degree of adaptation of the obturation material to the prepared root canal walls, with relatively low variability among samples.

### 3.1. Results of the One-Sample t-Test

**Figure 5:**
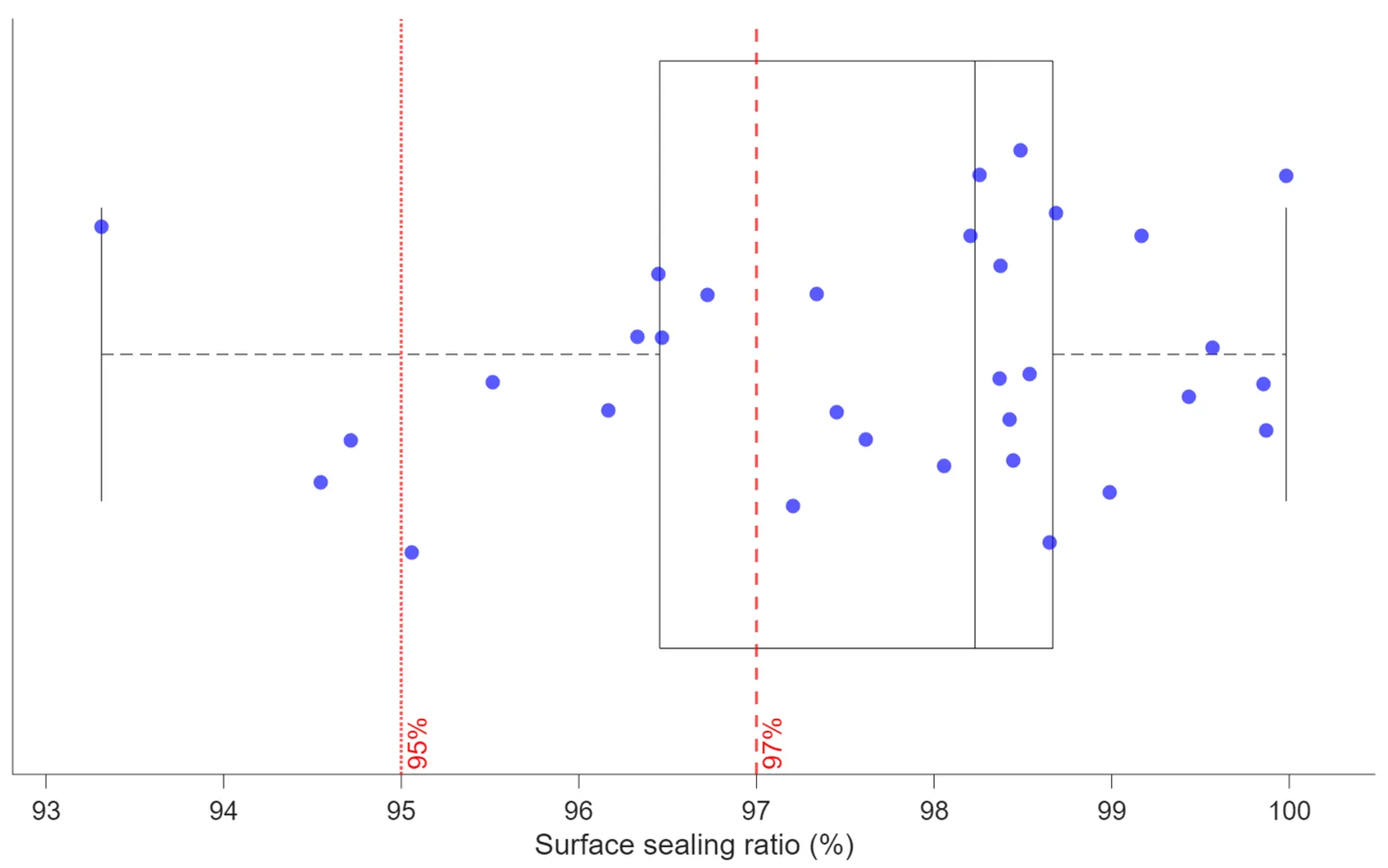
Horizontal box-and-scatter plot of distribution of the surface sealing ratio.

Two predefined exploratory analytical reference levels, 95% and 97%, were used to contextualize the distribution of the surface coverage values. Most measurements were close to or above 97%, whereas only a small number of values were below this level. The median was above 97%, and the interquartile range was narrow, indicating low dispersion of the data. The 95% reference level was located near the lower end of the distribution (Figure 5).

The box-and-scatter plot demonstrated a compact distribution of values without marked outliers, consistent with the low standard deviation. Overall, the data showed a compact distribution, with most values located in the upper range of surface coverage.

**Figure 6:**
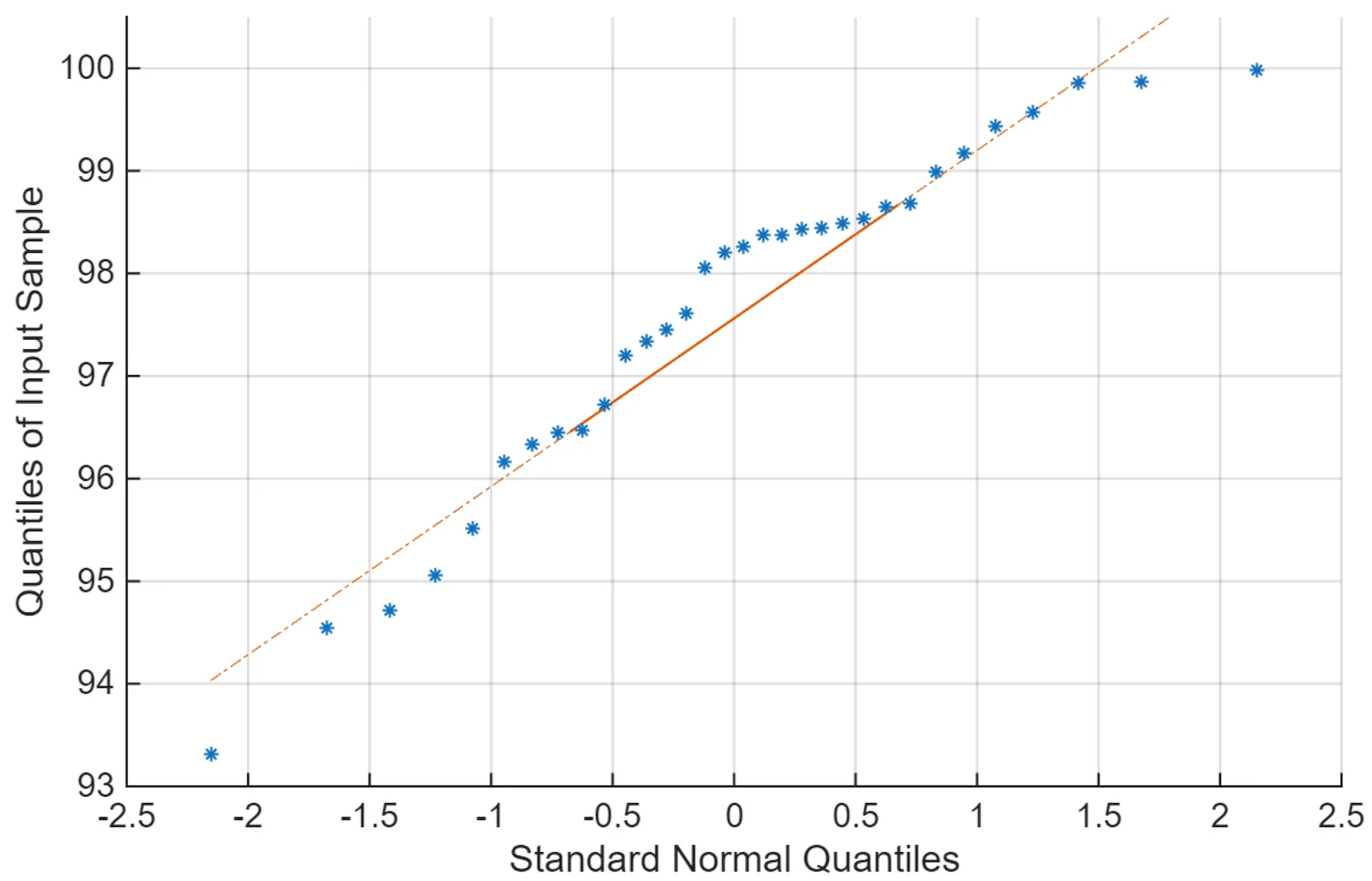
Q-Q plot of distribution of the surface sealing ratio.

A quantile–quantile (Q-Q) plot was used to assess whether the measured surface coverage values approximated a normal distribution (Figure 6).

Most data points were closely aligned with the reference line, particularly in the central part of the distribution, indicating that the assumption of approximate normality was acceptable. Minor deviations were observed at the lower and upper tails, which may be explained by the lower boundary of the minimum observed value and the upper limit of percentage data close to 100%. Overall, the Q-Q plot supported the use of a parametric test such as the one-sample *t*-test.

### 3.2. Statistical Results of the One-Sample t-Test in Relation to the a Priori Power Analysis

Based on pre-study assumptions the expected difference of approximately 4% and a standard deviation of 5.71% resulted in an estimated effect size of d = 0.7 for 29 root canals. For the final study design, a reference effect size of d = 0.66 was assumed for 32 canals with α = 0.05 and a power of 95%.

For the 95% exploratory reference level, the observed effect size (d = 1.55) was large, reflecting the difference between the measured mean surface coverage and this predefined analytical level. For the 97% exploratory reference level, the observed effect size (d = 0.37) was smaller, which is consistent with the limited difference between the measured mean and this higher reference level.

Overall, the findings were consistent with the a priori power analysis. The sample size of 32 canals was sufficient to detect medium to large effects with high statistical power, whereas smaller differences, such as those observed relative to the 97% reference value, should be interpreted more cautiously.

Within the limitations of this exploratory analysis, the mean surface coverage was above the 95% reference level and close to the 97% reference level.

The 95% and 97% values were retained only as exploratory analytical reference levels and not as clinically validated thresholds. Their purpose was to help describe how the measured values were distributed in relation to high levels of surface coverage. Therefore, the statistical comparisons should be interpreted as supportive descriptive analyses rather than as evidence that the material meets a clinically established performance criterion.

Table 1 presents the statistical results obtained for predefined exploratory surface coverage reference levels using the two-tailed one-sample *t*-test.

For the 95% exploratory analytical reference level, the one-sample *t*-test demonstrated that the mean surface coverage observed in the study was statistically higher than the predefined analytical value of 95% (t(31) = 8.78, *p* = 6.51 × 10^−10^). The measured mean surface coverage was 97.63% (SD = 1.70%), representing an average increase of 2.63 percentage points relative to the 95% reference level. This difference indicates that, within the analyzed sample, the extent of surface coverage consistently exceeded the lower exploratory analytical reference value selected for descriptive and comparative purposes.

Additional support for this observation is provided by the 95% confidence interval for the mean, which ranged from 97.02% to 98.24%. Because the entire confidence interval lies above the 95% reference level, the observed data suggest that the population mean surface coverage under the present ex vivo experimental conditions is likely to be greater than 95%. In practical terms, the confidence interval indicates that even the lower bound of the estimated mean remains above the predefined analytical reference value, reinforcing the statistical finding obtained from the one-sample *t*-test.

The magnitude of the observed difference was further assessed using Cohen’s d, which yielded a value of 1.55. According to conventional interpretations of effect size, this represents a large effect, indicating that the difference between the observed mean surface coverage and the 95% analytical reference level was substantial relative to the variability of the measurements within the sample. This effect size reflects the strength of the separation between the sample mean and the predefined analytical value and provides complementary information beyond statistical significance alone.

For the 97% exploratory analytical reference level, the observed mean surface coverage was 97.63% (SD = 1.70%), corresponding to an average difference of 0.63 percentage points above the predefined analytical value of 97%. To explore the relationship between the measured values and this reference level, a one-sample *t*-test was performed, yielding t(31) = 2.11 and *p* = 0.043. Because the analysis was conducted using a predefined significance level of α = 0.03, the null hypothesis was not rejected. Consequently, the statistical analysis did not provide sufficient evidence to conclude that the mean surface coverage differed from the 97% reference level under the selected analytical conditions.

The estimated 95% confidence interval for the mean surface coverage was [96.95%; 98.31%]. Importantly, this interval included the predefined 97% reference value, indicating that the true population mean compatible with the observed data could plausibly be slightly below, equal to, or slightly above 97%. The inclusion of the reference value within the confidence interval therefore suggests that the observed measurements were positioned in the vicinity of this exploratory analytical reference level. However, the confidence interval should not be interpreted as evidence that the mean is equivalent to 97%, nor does it establish that the observed values satisfy any predefined criterion of similarity.

The calculated effect size was small (Cohen’s d = 0.37), reflecting the relatively modest magnitude of the difference between the observed mean and the 97% reference value. From a descriptive perspective, this finding indicates that the average surface coverage measured in the present sample was numerically close to the selected exploratory reference level. Nevertheless, the effect size should be interpreted only as a measure of the magnitude of the observed difference and not as evidence supporting equivalence or interchangeability.

Overall, these findings indicate that the mean surface coverage obtained in this ex vivo investigation was located near the predefined 97% exploratory analytical reference level. The absence of a statistically significant difference at the chosen significance level does not demonstrate equivalence, similarity, non-inferiority, or compliance with a validated clinical threshold. Demonstrating such claims would require a different statistical framework, including the prospective definition of equivalence or non-inferiority margins and the application of dedicated methods such as the Two One-Sided Tests (TOST) procedure. Because no such margins were predefined and no formal equivalence or non-inferiority testing was performed, the present results should be regarded solely as descriptive and exploratory, reflecting the proximity of the observed mean surface coverage to the selected analytical reference value under the experimental conditions of this study.

## 4. Discussion

Within the limitations of this descriptive ex vivo study, the results indicate a high degree of adaptation of the novel filling material, Odne^®^Fill, to the root canal wall. The mean surface coverage was 97.63%, with a relatively small standard deviation, suggesting low variability among the samples. The results indicate that high surface coverage values were achieved consistently under the experimental conditions of this study. These results refer only to the measured surface coverage parameter and do not allow conclusions regarding superior sealing ability or clinical effectiveness.

High values of surface adaptability of the root canal filling material have been associated with a better sealing of the root canals. A homogenous, void-free and well adapted root canal filling is associated with a higher success rate of the endodontic treatment [27].

The surface coverage values were clustered within a narrow range with most measurements close to or above 97%. Limited areas of incomplete wall contact may be explained by anatomical complexity, local canal irregularities or methodological factors related to the image acquisition and segmentation process. The micro-CT investigation technique has been used to assess the quality of root canal fillings more accurately than periapical radiographs.

The voxel size used in this study ranged from 4.978 to 7.122 µm below values used otherwise to detect irregularities or voids in root canal filling materials [28].

This observational study describes the adaptability of a novel filling material to the root canal wall without employing a control group. The findings should be interpreted as reflecting the performance of the material under laboratory conditions and not as direct evidence of clinical sealing ability.

The novel filling material evaluated in the present study is an injectable light-curing hydrophilic material with water-like consistency before curing. It can be used in most of the canal anatomies regardless the used mechanical preparation method [22].

Minimal invasive mechanical instrumentation and even a non-instrumentation technique have already been described [29,30] but the filling of such canal geometries remains clinically a very challenging procedure.

For statistical interpretation, the measured values were compared with two predefined exploratory analytical reference levels of 95% and 97%. These values were not considered clinically validated thresholds, but were used only to provide a quantitative context for the observed distribution of surface coverage values. Relative to the 95% reference level, the observed mean surface coverage was higher, and the confidence interval was entirely above this value. In contrast, the comparison with the 97% reference level showed that the observed mean was close to this value, with only a small difference between the sample mean and the reference level.

These findings indicate that the material achieved high surface coverage under the present ex vivo experimental conditions. However, this should not be interpreted as proof of superior sealing ability or clinical effectiveness. Surface adaptation assessed by micro-CT represents only one aspect of the obturation quality. Other relevant properties, such as void distribution, dimensional stability, retreatability, resistance to leakage, and long-term behavior, were not assessed in the present study and should be addressed in further studies.

The results should also be interpreted in light of the study design. This was a single-arm laboratory investigation without a comparator group. Therefore, no conclusions can be drawn regarding superiority or equivalence in comparison with established obturation techniques. In addition, the use of extracted teeth and a manufacturer-specific preparation and irrigation protocol may limit transferability to routine clinical conditions.

Another point that merits consideration is the statistical approach. The predefined 95% and 97% values were used as exploratory analytical reference levels and not as clinically validated thresholds. Moreover, failure to detect a statistically significant difference from the 97% reference level should not be interpreted as statistical equivalence. Demonstrating equivalence or non-inferiority would require predefined margins and a dedicated statistical methodology, such as TOST, which was not performed in the present study.

The relatively small standard deviation confirms that the segmentation outcomes are not dominated by a few extreme values but rather reflect a stable and reproducible filling behavior.

The observed proximity of the measured surface coverage values to the selected reference levels merely indicates the extent to which the material adapted to the canal walls within the experimental conditions of the present study. Such findings provide useful information regarding the physical behavior of the material and its adaptation characteristics but do not allow conclusions regarding sealing effectiveness under clinical conditions, resistance to leakage, long-term dimensional stability, prevention of reinfection, periapical healing, or overall treatment outcomes. Furthermore, because the study was conducted under controlled laboratory conditions using an ex vivo model, the results cannot fully reproduce the biological, anatomical, and procedural variability encountered in clinical practice.

Therefore, the principal finding of the present study remains that Odne^®^Fill demonstrated a high degree of canal wall adaptation and extensive surface coverage in the evaluated root canals as assessed by micro-computed tomography. While these findings suggest favorable adaptation characteristics, their clinical significance cannot be inferred from the present data alone. Additional investigations, including comparative studies against established obturation materials and techniques, leakage and durability assessments, as well as prospective long-term clinical studies, are necessary to determine whether the observed adaptation characteristics translate into meaningful clinical benefits and improved treatment outcomes.

## 5. Conclusions

Overall, the present findings demonstrate that the investigated material achieved a high degree of canal wall adaptation in this ex vivo model. The exploratory comparisons with the predefined 95% and 97% analytical reference levels should be interpreted only as descriptive statistical analyses and not as an evidence of clinical effectiveness, equivalence, or superiority to the accepted root canal filling materials and procedures. The conclusions of this study are limited because a control group was not included in this descriptive study [31]. These results support further investigation of the material, particularly in comparative studies including established obturation techniques and additional outcome measures that are more directly related to clinical performance. Further investigations are also suggested to evaluate this obturation system in comparison with conventional techniques and assess the long-term stability and durability of the novel filling material under clinically relevant conditions.

## Figures and Tables

**Table 1:** Statistical results of one-sample *t*-tests for predefined exploratory surface coverage reference levels.

Parameter	95% Reference Level	97% Reference Level
Sample size (*n*)	32	32
Significance level (α)	0.05	0.03
Decision (h)	1	0
*p*-value	6.51 × 10^−10^	0.043
Mean surface ratio (%)	97.63	97.63
Standard deviation (%)	1.70	1.70
Degrees of freedom (df)	31	31
t-statistic	8.78	2.11
Confidence interval of mean (%)	[97.02; 98.24]	[96.95; 98.31]
Cohen’s d (effect size)	1.55	0.37

## Data Availability

The data resulted from this research project is at this moment the only one available as stated in this article.
